# Performance of (Instrumental) Activities of Daily Living and Physical Capacity in Spanish Adults with Intellectual Disabilities: A Cross-Sectional Pilot Study

**DOI:** 10.3390/healthcare9040435

**Published:** 2021-04-08

**Authors:** Laura Delgado-Lobete, Rebeca Montes-Montes, Carlos Freire, María del Mar Ferradás

**Affiliations:** 1 Health Integration and Promotion Research Unit (INTEGRA SAÚDE), Faculty of Health Sciences, University of A Coruña, 15011 A Coruña, Spain; l.delgado@udc.es; 2 TALIONIS Research Group, Research Centre of the Galician University System, Centre for Information and Communications Technology Research (CITIC), Universidade da Coruña, 15008 A Coruña, Spain; 3 Centro Ocupacional Pascual Veiga, 15005 A Coruña, Spain; 4 Department of Psychology, University of A Coruña, 15071 A Coruña, Spain; carlos.freire.rodriguez@udc.es (C.F.); mar.ferradasc@udc.es (M.d.M.F.)

**Keywords:** intellectual disability, activities of daily living, physical fitness, daily functioning

## Abstract

Performance in basic and instrumental activities of daily living (ADL; IADL) is an essential indicator of daily functioning and health of people with intellectual disabilities (ID). The aims of this pilot study were to describe the profile of ADL and IADL performance in Spanish adults with ID, and to examine its association with functional physical skills. The Waisman Activities of Daily Living Scale for adolescents and adults with developmental disabilities (W-ADL) scale was administered to the caregivers of twenty adults with ID (mean age = 41.0, SD = 10.1; women = 75.0%). In addition, dynamic balance and maximum walking speed (MWS), lower-body strength, aerobic capacity and manual dexterity of participants were individualized assessed. The results showed that 40% of adults with ID were completely independent in ADL, but all participants reported activity limitations in at least one IADL. Dynamic balance and MWS, lower-body strength and manual dexterity showed significant and moderate-to-strong correlations with daily functioning (*r* = 0.495–0.814; *p* < 0.05). Linear regression analysis indicated that lower-body strength and manual dexterity significantly predicted activity performance in adults with ID (adjusted R^2^ = 0.816, *p* = 0.004–0.016). This study highlights the need to support the performance of both ADL and IADL and to promote physical fitness in Spanish community-based care centers for adults with ID.

## 1. Introduction

Intellectual disabilities (ID) are a group of early onset disabilities characterized by significant limitations in both intellectual functioning (i.e., general mental capacity) and in culturally appropriate adaptative behavior, including those conceptual, social and practical skills involved in everyday performance [1,2]. Estimates indicate that approximately 1% of general population present with ID worldwide, with higher prevalence rates found in low- and middle-income countries [3,4]. According to the most recent data available, the prevalence of intellectual and developmental disabilities in the Spanish population ranges from 0.7% to 0.9% [5], which is in line to the mean prevalence rate in Western Europe [4].

People with ID experience different levels of limitations in basic aspects necessary for independent functioning and living. Satisfactory daily functioning is recognized as a main component of health status and well-being by the World Health Organization’s International Classification of Functioning (ICF) [6]. According to the ICF, both capacities, performance and participation are essential parts of functioning. For instance, impairments in capacity (i.e., an individual’s ability to carry out a task or action) may limit the performance of activities (i.e., what the person actually does in his or her daily environment), which will in turn restrict his or her participation (i.e., involvement in significative life situations) [6]. In addition, personal and environmental factors may mediate or moderate this relationship [6], which is consistent with previous theoretical frameworks in Occupational Therapy, such as the Person–Environment–Occupation and the Ecology of Human Performance models [7]. Moreover, this interrelated and multifactorial model of functioning was further explored and confirmed for people with ID specifically in the model of human functioning proposed by the American Association of Intellectual and Developmental Disabilities (AAIDD) [8].

There are two main occupations oriented towards self-care and self-maintenance within the home and community: activities of daily living (ADL; also referred to as basic ADL), and instrumental activities (IADL) [9]. While ADL describes personal activities such as bathing and grooming, toileting, dressing or eating, ADL involves those activities that support daily life within the home and community, such as meal preparation and cleanup, home management or shopping [9,10]. The independent performance of both ADL and IADL is an essential indicator of functioning, health status and all-cause mortality in adults with ID [6,8,11,12], but this population usually encounters significant activity limitations [12,13,14]. Functional physical skills such as manual dexterity, balance, walking speed, muscular strength and cardiorespiratory fitness are important predictors of daily functioning in individuals with ID [15,16], and contribute to increasing survival in this population [17]. Furthermore, poor physical fitness is a main predictor for a decline in both ADL and IADL performance in adults with ID, and it seems to play a role as relevant as cognitive abilities in daily functioning of people with ID [15,16].

In Spain, up to 44% of people with ID shows mild, moderate or severe dependency in basic daily functioning [5]. In addition, Spanish adults with ID display sedentary patterns and poor physical fitness, which further increase the risk of poor health status in this population [18,19]. It has been advised to increase research on activity limitations among adults with ID using validated measurement tools that can inform research and tailored community health policies [13,20]. However, to the best of our knowledge, there is no study that explores limitations in both ADL and IADL performance in Spanish adults with ID, nor its association with functional physical abilities.

The objectives of this study were: (a) to explore the profile of ADL and IADL performance in Spanish adults with ID; and (b) to examine the relationship between the performance of daily activities and functional physical skills. Based on previous research, we hypothesized that functional physical skills would be associated with daily functioning in adults with ID.

## 2. Materials and Methods

### 2.1. Research Ethics, Participants and Procedure

We conducted a cross-sectional pilot study. Ethical approval was granted by the Autonomic Research Ethics Committee of Galicia. Informed consent was obtained from all participants and/or their legal guardians. Participants were eligible for the study if they: (a) had a diagnosis of mild to moderate ID; (b) were able to walk independently; (c) understood and were willing to perform all the functional measurements; and (d) the individual and/or their legal guardian (i.e., parents or siblings) provided informed consent. Participants were excluded if they were unable to perform the functional physical tests safely.

The sample size of this pilot study was calculated on the main aim of the research (i.e., to explore daily performance of Spanish adults with ID). A sample of 20 participants was sufficient to provide preliminary data on daily performance as measured by the W-ADL (confidence level = 99%; precision = 5%), considering that the reported variance of the W-ADL ranges from 30.3 to 59.3 in adults with mild to severe ID [20].

Data regarding a convenience sample of 20 adults with ID was included. Participants attended a state-funded occupational health care center in A Coruña (Spain). Of the 24 individuals attending the center, 1 was excluded of the study because they needed assistance to walk, and 3 caregivers refused to participate. Of the 20 participants included in the study, 5 had Down syndrome and 15 had ID due to another cause (i.e., infant epilepsy, neonatal head trauma or idiopathic intellectual disability). Table 1 shows the sociodemographic characteristics of the sample.

The main caregivers of the participants filled in the Waisman Activities of Daily Living Scale for adolescents and adults with developmental disabilities (W-ADL) as part of the occupational therapy assessment at the occupational health care center.

Physical fitness tests were part of the physical fitness assessment of the occupational health center and were performed in a fixed sequence. All assessments were individually guided by an occupational therapist and a physical activity instructor to ensure the reliability and safety of the physical fitness tests. The two test observers had experience the in working with adults with IDD. The participants performed the three physical tests on two days in the same week that their legal guardians completed the W-ADL. Dynamic balance and maximum walking speed (MWS), and lower-body strength, were measured on the first day, and aerobic endurance was measured on the second day. Before each assessment, the test was demonstrated twice by the test observers (i.e., occupational therapist and physical activity instructor) and instructions were provided to ensure that the participants understood the task. Finally, manual dexterity was individually measured in a separate session by the same occupational therapist who observed the physical tests.

### 2.2. Measurements

#### 2.2.1. Performance of Activities of Daily Living

The W-ADL was used to measure activity performance limitations of participants [20]. The W-ADL is a caregiver-report instrument that comprises seventeen activities, including six ADL (e.g., washing/bathing, grooming, brushing teeth and hair, dressing) and eleven IADL (e.g., doing household tasks, preparing food, shopping in stores). Caregivers rate how the person with IDD performs each task on a three-point Likert scale (0 = does not do at all, 1 = does with help, 2 = independent or does on own), where higher scores indicate better performance. The W-ADL is based on the ICF framework and it has been validated for use as a caregiver-report measure with adults with a variety of IDD, including people with Down syndrome and other ID, Autism Spectrum Disorder and Fragile-X syndrome. Its original internal consistency, the test–retest and inter-rater values for people with ID were excellent (Cronbanch’s alpha = 0.91–0.94; weighted kappas (test–retest reliability) = 0.92–0.93; weighted kappa (inter-rater reliability) = 0.88) [20]. The W-ADL was independently translated into European Spanish by two Spanish occupational therapists fluent in English, and then synthetized into one single translation following international recommendations [21]. This version was then confronted with three professionals working with adults with ID and three caregivers of adults with ID to ensure comprehensibility and cultural equivalence of the translated W-ADL. The internal consistency value of the W-ADL for the sample of this study was good (Cronbach’s alpha = 0.82).

#### 2.2.2. Functional Physical Tests

A modified version of the timed up and go test (TUGT) was used to evaluate dynamic balance and MWS. Participants were asked to rise from an armless chair without using the arms, run or walk as fast as they could to a pylon at 3 m in distance, come back again as fast as they could and go back to the chair sitting again. Three trials were performed with a 5 min rest between trials, and the best record (i.e., the shortest time) was recorded for the analysis. The TUGT is a reliable measure to assess mobility in people with ID [22,23] and has been previously used in adults with ID [24,25].

Lower-body strength was measured using the 30-s chair stand test (CST), which is a frequently used test to evaluate lower-body muscle strength and endurance [26]. Participants were asked to sit down and stand upright (full stance) as fast and often as possible in 30 s with arms folded across the chest (i.e., without using hands). Participants repeated this test three times with a 5 min rest between trials, and the best of these three trials (i.e., the maximum numbers of repetitions) was recorded. The CST shows acceptable feasibility and good short-time test–retest reliability in adults with ID (ICC = 0.72) [27], and it has been previously used in this population [28,29].

In order to evaluate aerobic capacity, the participants performed a modified 2-min step test (TMST) [30]. The participants were asked to march in place as fast as possible for 2 min while lifting the knees to their iliac crest when standing. Participants were allowed to put their hands on a surface in front of them during the test, and visual marks were placed at the required lifting height for each participant. A higher number of repetitions indicated a better aerobic capacity. This test was only performed once to avoid fatigue bias. The TMST has been previously used in adults with ID [31].

The nine-hole peg test (NHPT) was used to assess manual motor dexterity [32]. The NHPT is a widely used, reliable and validated measure of finger and hand dexterity. Participants were asked to repeatedly place and then remove nine pegs into nine holes, one at a time, as quickly as possible, without any assistance of the other hand. The seconds needed to complete the NHPT indicate the level of manual dexterity (i.e., more time to complete the task indicates poorer dexterity). After one trial with each hand, only the score of the participants’ dominant hand was included in the analyses. The NHPT is easy to understand and to administer and thus it is feasible to use it in adults with ID [33].

### 2.3. Data Analysis

Descriptive data are presented as numbers and proportions. Due to the small sample size (<30), normal distribution of the data was explored using the Shapiro–Wilk test [34]. Descriptive statistics of the W-ADL and the functional tests were calculated as mean and standard deviation (SD) for normally distributed variables and as median and interquartile rank (IQR) for non-normal distributions. Ability to perform each activity of the W-ADL was calculated as percentage according to the level of activity limitations for each item. Association between daily performance and functional capacities was explored with the Pearson or Spearman correlation coefficients for normal and non-normal distributed data, respectively. Finally, we performed a linear regression model (stepwise method) to determine which functional abilities (i.e., dynamic balance and MWS, lower-body strength, aerobic capacity and manual dexterity) better predicted daily performance in adults with ID. Only those functional abilities that previously showed a significant association with performance were included in the regression analysis. Assumptions of normality, linearity, homoscedasticity, and absence of multicollinearity were confirmed [35]. In addition, the adjusted R-square was explored to determine the explanatory power of the model.

The sample size was estimated using EPIDAT v. 3.1 for Windows (Consellería de Sanidade & Organización Panamericana de la Salud-World Health Organization, Galicia, Spain). Statistical analyses were performed using IBM SPSS v. 25 for Windows (SPSS Inc., Chicago, IL, USA).

## 3. Results

Overall, participants reported more activity limitations in IADL performance (Table 2). Most of participants did not have severe activity limitations in any ADL, but only 40% of participants were independent in all ADL. Regarding the performance of instrumental activities, all participants reported mild or severe activity limitations in at least one IADL.

Descriptive statistics of the W-ADL and the functional tests for participants with mild and moderate-or-severe ID are displayed in Table 3. Scale correlations between the W-ADL and the functional tests for total sample are shown in Table 4. Daily performance showed a significant and moderate-to-strong correlation with dynamic balance and MWS, lower-body strength and manual dexterity, but not with aerobic capacity (*r* = 0.495–0.814; *p* < 0.05).

A stepwise linear regression analysis was conducted entering daily performance as the dependent variable and dynamic balance and MWS, lower-body strength and manual dexterity as predictors. The model summary and coefficients are shown in Table 5 and Table 6. The final model included lower-body strength and manual dexterity as predictors of performance, and it explained 81.6% of the variability of performance in ADL.

## 4. Discussion

Independent performance of basic and instrumental daily activities is important to health and functioning of ageing adults with ID [11,12,14]. We aimed to preliminary explore the performance in ADL and IADL of Spanish adults with ID and to study its relationship with physical capacity.

The findings from this pilot study revealed that 40% of participants were totally independent for ADL performance. This is consistent with previous studies that showed that complete independent performance of basic ADL in middle-aged and older adults with ID ranges between 14.5% and 49.8% depending on cause and severity of ID, age, health status and physical fitness [13,14,15,16]. In addition, all participants were completely able to drink and eat on their own, and 80–90% were independent in dressing/undressing and toileting performance. Conversely, caregivers of adults with ID in this study reported a high prevalence of activity limitations in washing, bathing or grooming. Similar results were found by Lin et al. [13], who reported that 80.6% of middle-aged adults with ID in Taiwan were able to feed themselves on their own, but all participants reported that they needed some level of assistance to perform bathing or grooming activities. Adults with ID have been reported to need more help in washing or dressing activities than in feeding-related activities independently of the severity of ID or living arrangement [11,36]. Even though all ADL are oriented toward taking care of one’s own body, the demands of each activity vary according to actions and performance skills, body functions and structures, sequencing and timing or objects used [9], which contributes to explaining the high variability in ADL performance in this population. For instance, washing, bathing or grooming activities involve more complex and sequenced tasks, and require more demands from different body functions and structures, than feeding-related activities.

Regarding the performance of instrumental activities, all of the participants in this study showed some level of activity limitation in at least one IADL according to their caregivers. Data on IADL performance in this population is scarce, but research indicates that fewer than 3% of older adults with ID are completely independent in IADL [14,16]. For young adults with mild or severe ID (<25 years old), the prevalence of limitations in at least one IADL range between 47.5% and 96.7% [37]. Findings from longitudinal studies show that ability to perform IADL decreases very quickly in ageing adults with ID [16]. Overall, people with ID report significantly lower abilities to perform IADL in comparison to ADL independently of age, level of ID, cultural context or place of residence [14,15,16,36,37]. The extremely high prevalence of activity limitations in IADL performance found in this sample could contribute to explain the differences on the W-ADL total score between Maenner et al. research [20] and our study, as it seems that performance of ADL is quite similar to that reported in the literature.

It is to be expected that people with developmental disabilities encounter more difficulties in IADL as they involve more complex tasks in terms of sequency and organizing [9,10]. While the assessment of the specific IADL depends on each measure, research suggests that certain IADLs are particularly difficult to perform for adults with ID. For instance, only two participants of this study were reported to be able to manage money on their own or with help, only one participant was able to do laundry, and none of the participant were able to prepare a complete meal, while most participants either needed help or were unable to mix and cook simple foods. Conversely, most participants were able to make their own bed independently or with help or do light household tasks. The ability to handle finances, to do laundry or to plan and prepare a complete meal are particularly challenging activities for adults with ID [36,37]. Those are remarkably complex IADL that demand several cognitive, process and organizing skills and which require practice and training [9]. However, it is possible that adults with ID do not have many opportunities to perform and to master those IADL on their daily basis. Most people with intellectual or developmental disabilities in Spain either attend day and day-and-night care centers or they live in residential settings, while only 6.5% receive in-home resources aimed to promote autonomy and daily functioning [38]. It has been argued that place of residence or care center may reduce potential participation and performance of certain IADL [36]. For instance, lunch meals are already prepared when they are distributed to people attending occupational health centers, day care centers or residential settings in Spain, which means that clients with ID do not need to plan or prepare complete meals or do grocery shopping, and thus they have fewer opportunities to master these daily living skills.

Overall, the mean and SD scores on the total W-ADL scale in this study were lower than those reported by Maenner et al. for North American adults with mild, moderate and severe ID [20], which may suggest that Spanish adults with ID face more activity limitations. Daily performance is influenced by personal, environmental, family and cultural factors [6,8,9], and therefore it is important to identify potential difficulties in the performance of specific basic and instrumental activities by people with ID living in different contexts.

The second aim of this study was to explore the association between daily functioning, defined as performance in ADL and IADL, and functional physical abilities in adults with ID. We found that, in Spanish adults with ID, dynamic balance and MWS, lower-body strength and manual dexterity were associated with performance of ADL and IADL as reported by caregivers, but only lower-body strength and manual dexterity predicted daily functioning. These results further support the hypothesis that low physical fitness plays a relevant role in the ability to perform ADL and IADL in ageing people with ID [15,16,39]. For instance, the loss of muscle mass and strength in the lower-body has been associated with poor ADL functioning in young and older adults with ID [31,39]. In addition, findings from longitudinal studies on older adults with ID show that decline in independent performance of both ADL and IADL is directly influenced by mobility and physical fitness, including manual dexterity, balance, fast and safe gait, cardiorespiratory capacity and muscle endurance, even after correcting for other determinant characteristics such as sex, age and level of ID [15,16]. Addressing daily functioning in this population needs to have priority in public health policy, given that adults with ID show extremely poor physical fitness [28] and are prone to premature ageing [40].

The high prevalence of activity limitations reported in the present study suggests the need for increased support for Spanish people with ID and their families. Occupational Therapy interventions should be extensively available to this population in Spain in order to support satisfactory performance and functioning. In addition, lower-body strength, dynamic balance, cardiorespiratory endurance and manual dexterity are necessary skills to perform most basic and instrumental daily activities. It is necessary that interventions aimed at promoting daily functioning in individuals with ID include the training of functional physical skills, which has been proven to be effective in this population [24,33,41].

### Strengths, Limitations and Implications for Future Research

The strengths of this study include the use of a measure specifically designed to evaluate performance in adults with intellectual and developmental disabilities, and the use of clinically oriented and simple tests to accurately assess physical abilities in individuals with ID. In addition, our results provide preliminary evidence supporting the assessment and promotion of functional physical capacities in adults with ID attending day care centers in Spain. However, several limitations must be addressed. First, the study design does not allow for establishing causality between physical abilities and daily functioning. Longitudinal studies could be conducted to investigate physical- and disability-related factors associated with the decline of daily performance in Spanish individuals with ID.

Second, a number of limitations regarding sample size need to be considered. This study aimed to provide preliminary data to fill the gap on research on daily functioning in Spanish adults with ID using a comprehensive assessment in a limited sample size. Although the sample size was estimated to provide preliminary reliable data on activity performance based on previous studies on international populations, it was not sufficient enough to allow for exploring statistical differences in performance and physical skills between people with mild, moderate or severe ID. Moreover, the generalization of the results to other Spanish populations with ID is restricted as the sample was recruited from a single occupational health care center, and thus adults with ID living in nursing homes or attending day-and-night care centers may display different activity performance limitations. In addition, the limited sample size may introduce bias in the bivariate and multivariate analyses, which should be taken into account when interpreting the statistical models. Thus, the results from this study should be further confirmed in future studies. However, the findings of this paper are valuable as they provide a relevant starting-point for future research on daily functioning involving larger samples of adults with ID and different living conditions and clinical characteristics. Likewise, the descriptive and inferential results of this study may contribute to inform the sample size calculations and feasible assessment strategies of larger cohorts of Spanish adults with ID.

Additionally, our results indicate that the W-ADL is a feasible and reliable measure to assess activity performance limitations in Spanish adults with mild, moderate and severe ID. Thus, clinicians and researchers can use this freely available and easy to use instrument to quickly asses daily functioning of adults with intellectual and developmental disabilities in Spain, which can support tailored interventions aimed to promote satisfactory performance in those ADL and IADL that are more challenging for this population.

## 5. Conclusions

Spanish adults with ID face activity limitations in a high range of basic and instrumental daily activities. Participants reported more difficulties in performance of IADL than in performance of ADL. In addition, lower-body strength and manual dexterity were significantly related to performance of ADL and IADL. As people with intellectual and developmental disabilities reach a higher life expectancy, community health policies need to be prepared to meet the needs of this population and their families and to promote independent and satisfactory daily performance and physical health of people with ID.

## Figures and Tables

**Table 1:** Sociodemographic characteristics of the sample (*n* = 20).

Sociodemographic Variables	*N*
Age (range)	22–62
Age (mean (SD))	41.0 (10.1)
Sex (*n* (%))	
Women	15 (75.0)
Men	5 (25.0)
Grade of documented disability (*n* (%)) ^a^	
Mild disability	13 (68.4)
Moderate disability	6 (31.6)
Level of intellectual disability (*n* (%))	
Mild	10 (50.0)
Moderate or severe	10 (50.0)

SD = standard deviation, ^a^ = data available for 19 participants.

**Table 2:** Performance of basic (ADL) and instrumental activities of daily living (IADL).

Activities	Independent or Does on Own *N* (%)	Does with Help *N* (%)	Does Not Do at All *N* (%)
Making own bed ^a^	8 (40.0)	9 (45.0)	3 (15.0)
Doing household tasks ^a^	4 (20.0)	13 (65.0)	3 (15.0)
Doing errands ^a^	2 (10.0)	11 (55.0)	7 (35.0)
Doing home repairs ^a^	0 (10.0)	2 (10.0)	18 (90.0)
Doing laundry ^a^	1 (5.0)	8 (40.0)	11 (55.0)
Washing/bathing ^b^	10 (50.0)	8 (40.0)	2 (10.0)
Grooming, brushing teeth/hair ^b^	11 (55.0)	9 (45.0)	0 (0.0)
Dressing/undressing ^b^	16 (80.0)	4 (20.0)	0 (0.0)
Toileting ^b^	18 (90.0)	2 (10.0)	0 (0.0)
Preparing simple foods ^a^	10 (50.0)	7 (35.0)	3 (15.0)
Mixing and cooking simple foods ^a^	7 (35.0)	4 (20.0)	9 (45.0)
Preparing complete meal ^a^	0 (0.0)	2 (10.0)	18 (90.0)
Setting/clearing table ^a^	13 (65.0)	6 (30.0)	1 (5.0)
Drinking from a cup ^b^	20 (100.0)	0 (0.0)	0 (0.0)
Eating from a plate ^b^	20 (100.0)	0 (0.0)	0 (0.0)
Washing dishes ^a^	5 (25.0)	11 (55.0)	4 (20.0)
Banking/managing daily finances ^a^	1 (5.0)	1 (5.0)	18 (90.0)

^a^ = instrumental activity of daily living, ^b^ = activity of daily living.

**Table 3:** Descriptive statistics.

Measures	Total (*n* = 20)	Mild ID (*n* = 10)	Moderate or Severe ID (*n* = 10)
W-ADL, m (SD)	19.5 (5.7)	21.0 (5.0)	17.9 (6.2)
mTUGT, md (IQR)	5.9 (16.5)	5.9 (4.5)	5.9 (16.5)
CST, m (SD)	13.3 (4.8)	13.4 (4.0)	13.2 (5.6)
mTMST, m (SD)	52.4 (26.2)	45.8 (17.2)	58.3 (32.0)
NHPT, m (SD)	39.5 (12.3)	32.8 (7.0)	45.3 (13.3)

W-ADL = Waisman Activities of Daily Living Scale; mTUGT = modified timed up and go test; CST = 30-s chair stand test; mTMST = modified 2-min step test; NHPT = nine-hole peg test; m = mean; SD = standard deviation, md = median; IQR = interquartile rank.

**Table 4:** Correlation between daily functioning and functional abilities.

Measures	mTUGT	CSTT	mTMST	NHPT
W-ADL	−0.495 * ^a^	0.621 ** ^b^	0.390 ^b^	−0.814 *** ^b^
mTUGT	-	−0.734 *** ^a^	−0.723 *** ^a^	0.732 ** ^a^
CST	-	-	0.689 *** ^b^	−0.676 ** ^b^
mTMST	-	-	-	−0.482 ^b^

W-ADL = Waisman Activities of Daily Living Scale; mTUGT = modified timed up and go test; CST = 30-s chair stand test; mTMST = modified 2-min step test; NHPT = nine-hole peg test, * *p* < 0.05, ** *p* < 0.01; *** *p* < 0.001, ^a^ = Spearman correlation coefficient, ^b^ = Pearson correlation coefficient.

**Table 5:** Model summary (stepwise method).

Model	R	R^2^	Adjusted R^2^	Standard Error of the Estimate
1 ^a^	0.919	0.845	0.799	2.7
2 ^b^	0.919	0.845	0.816	2.6

^a^ = predictors: (constant), mTUGT, CST, NHPT, ^b^ = predictors: (constant), CST, NHPT.

**Table 6:** Model coefficients (stepwise method).

Model		Coefficient	SE	Lower Limit	Upper Limit	*p* Value
1	(Constant)	19.524	5.715	6.789	32.259	0.007
	mTUGT	−0.062	0.271	−0.666	0.543	0.825
	CST	0.680	0.236	0.154	1.205	0.016
	NHPT	−0.210	0.090	−0.412	−0.009	0.042
2	(Constant)	19.150	5.233	7.633	30.668	0.004
	CST	0.701	0.207	0.245	1.157	0.006
	NHPT	−0.219	0.077	−0.390	−0.049	0.016

SE = standard error; mTUGT = modified timed up and go test; CST = 30-s chair stand test; NHPT = nine-hole peg test.

## Data Availability

The data that support the findings of this study are available from the corresponding author, R.M.-M., upon reasonable request.

## References

[1] Schalock R.L., Luckasson R., Tassé M.J. (2021). Intellectual Disability: Definition, Diagnosis, Classification, and Systems of Supports.

[2] American Psychiatry Association (2013). Diagnostic and Statistical Manual of Mental Disorders.

[3] Maulik P.K., Mascarenhas M.N., Mathers C.D., Dua T., Saxena S. (2011). Prevalence of intellectual disability: A meta-analysis of population-based studies. Res. Dev. Disabil..

[4] Wittchen H.U., Jacobi F., Rehm J., Gustavsson A., Svensson M., Jönson B., Olensen J., Allgulander C., Alonso J., Faravelli C. (2011). The size and burden of mental disorders and other disorders of the brain in Europe 2010. Eur. Neuropsychopharmacol..

[5] Jiménez Lara A. (2019). Informe Olivenza 2019 Sobre la Situación General de la Discapacidad en España.

[6] World Health Organization (2001). International Classification of Functioning, Disability and Health: ICF.

[7] Turpin M., Iwama M.K. (2011). Using Occupational Therapy Models in Practice: A Field Guide.

[8] Wehmeyer M.L., Buntinx W.H.E., Lachapelle Y., Luckasson R.A., Schalock R.L., Verdugo M.A., Borthwick-Duffy S., Bradley V., Craig E.M., Coulter D.L. (2008). The intellectual disability construct and its relation to human functioning. Intellect. Dev. Disabil..

[9] American Occupational Therapy Association (2020). Occupational therapy practice framework: Domain and process fourth edition. Am. J. Occup. Ther..

[10] Katz S. (1983). Assessing self-maintenance: Activities of daily living, mobility, and instrumental activities of daily living. J. Am. Geriatr. Soc..

[11] Lifshitz H., Merrick J., Morad M. (2008). Health status and ADL functioning of older persons with intellectual disability: Community residence versus residential care centers. Res. Dev. Disabil..

[12] Oppewal A., Schoufour J.D., Evenhuis H.M., Festen D.A.M., Hilgenkamp T.I.M. (2016). Ouderen met een verstandelijke beperking verliezen veel zelfredzaamheid gedurende drie jaar: Resultaten van de Gezond Ouder studie (GOUD). Tijdschr. Gerontol. Geriatr..

[13] Lin L.-P., Hsia Y.-C., Hsu S.-W., Loh C.-H., Wu C.-L., Lin J.-D. (2013). Caregivers’ reported functional limitations in activities of daily living among middle-aged adults with intellectual disabilities. Res. Dev. Disabil..

[14] Hilgenkamp T.I.M., van Wijck R., Evenhuis H.M. (2011). (Instrumental) activities of daily living in older adults with intellectual disabilities. Res. Dev. Disabil..

[15] Oppewal A., Hilgenkamp T.I.M., van Wijck A., Schoufour J.D., Venhuis H.M. (2014). Physical fitness is predictive for a decline in daily functioning in older adults with intellectual disabilities: Results of the HA-ID study. Res. Dev. Disabil..

[16] Oppewal A., Hilgenkamp T.I.M., van Wijck R., Schoufour J.D., Evenhuis H.M. (2015). Physical fitness is predictive for a decline in the ability to perform instrumental activities of daily living in older adults with intellectual disabilities: Results of the HA-ID study. Res. Dev. Disabil..

[17] Oppewal A., Hilgenkamp T.I.M. (2019). Physical fitness is predictive for 5-year survival in older adults with intellectual disabilities. J. Appl. Res. Intellect. Disabil..

[18] Oviedo G.R., Travier N., Guerra-Balic M. (2017). Sedentary and Physical Activity Patterns in Adults with Intellectual Disability. Int. J. Environ. Res. Public Health.

[19] Oviedo G.R., Tamulevicius N., Guerra-Balic M. (2019). Physical Activity and Sedentary Time in Active and Non-Active Adults with Intellectual Disability: A Comparative Study. Int. J. Environ. Res. Public Health.

[20] Maenner M.J., Smith L.E., Hong J., Makuch R., Greenberg J.S., Mailick M.R. (2013). Evaluation of an activities of daily living scale for adolescents and adults with developmental disabilities. Disabil. Health J.

[21] Koller M., Kantzer V., Mear I., Zarzar K., Martin M., Greimel E., Bottomley A., Arnott M., Kulis D., Tca-Sig T.I. (2012). The ISOQOL TCA-SIG. The process of reconciliation: Evaluation of guidelines for translating quality-of-life questionnaires. Expert Rev. Pharm. Out..

[22] Blomqvist S., Wester A., Sundelin G., Rehn B. (2012). Test-retest reliability, smallest real difference and concurrent validity of six different balance tests on young people with mild to moderate intellectual disability. Physiotherapy.

[23] Salb J., Finlayson J., Almutaseb S., Scharfenberg B., Becker C., Sieber C., Freiberger E. (2015). Test-retest reliability and agreement of physical fall risk assessment tools in adults with intellectual disabilities. J. Intellect. Disabil. Res..

[24] Oviedo G.R., Guerra-Balic M., Baynard T., Javierre C. (2014). Effects of aerobic, resistance and balance training in adults with intellectual disabilities. Res. Dev. Disabil..

[25] Enkelaar L., Smulders E., van Schrojenstein Lantman-de Valk H., Weerdesteyn V., Geurts A.C.H. (2013). Clinical measures are feasible and sensitive to assess balance and gait capacities in older persons with mild to moderate Intellectual Disabilities. Res. Dev. Disabil..

[26] Rikli R.E., Jones C.J. (2013). Senior Fitness Test Manual.

[27] Hilgenkamp T.I.M., van Wijck R., Evenhuis H.M. (2012). Feasibility and reliability of physical fitness tests in older adults with intellectual disability: A pilot study. J. Intellect. Dev. Disabil..

[28] Hilgenkamp T.I.M., van Wijck R., Evenhuis H.M. (2012). Low physical fitness levels in older adults with ID: Results of the HA-ID study. Res. Dev. Diabil..

[29] Calders P., Elmahgoub S., Roman de Mettelinge T., Vandenbroeck C., Dewandele I., Rombaut L., Vandevelde A., Cambier D. (2011). Effect of combined exercise training on physical and metabolic fitness in adults with intellectual disability: A controlled trial. Clin. Rehabil..

[30] Rikli R.E., Jones C.J. (1999). Development and validation of a functional fitness test for community-residing older adults. J. Aging Phys. Activ..

[31] Cuesta-Vargas A.I., Pérez-Cruzado D. (2014). Relationship between Barthel index with physical tests in adults with intellectual disabilities. Springerplus.

[32] Oxford Grice K., Vogel K.A., Le V., Mitchell A., Muniz S., Vollmer M.A. (2003). Adult norms for a commercially available Nine Hole Peg Test for finger dexterity. Am. J. Occup. Ther..

[33] Cantone M., Catalano M.A., Lanza G., La Delfa G., Ferri R., Pennisi M., Bella R., Pennisi G., Bramanti A. (2018). Motor and Perceptual Recovery in Adult Patients with Mild Intellectual Disability. Neural. Plast..

[34] Ghasemi A., Zahediasl S. (2012). Normality Tests for Statistical Analysis: A Guide for Non-Statisticians. Int. J. Endocrinol. Metab..

[35] Vilà Baños R., Torrado Fonseca M., Reguant Álvarez M. (2019). Multiple regression analysis using SPSS Statistics: A practical example. REIRE.

[36] King E., Okodogbe T., Burke E., McCarron M., McCallion P., O’Donovan M.A. (2017). Activities of daily living and transition to community living for adults with intellectual disabilities. Scand. J. Occup. Ther..

[37] Van naarden Braun K., Yeargin-Allsopp M., Lollar D. (2009). Activity limitations among young adults with developmental disabilities: A population-based follow-up study. Res. Dev. Disabil..

[38] Díaz Velázquez E., Rubio Burgos S. (2020). Las Personas con Discapacidad Intelectual o del Desarrollo en el Sistema Para la Autonomía y Atención a la Dependencia (SAAD).

[39] Carmeli E., Imam B., Merrick J. (2012). The relationship of pre-sarcopenia (low muscle mass) and sarcopenia (loss of muscle strength) with functional decline in individuals with intellectual disability (ID). Arch. Gerontol. Geriatr..

[40] Berjano Peirats E., García Burgos E. (2010). Discapacidad Intelectual y Envejecimiento: Un Problema Social del Siglo XXI.

[41] Zurita-Ortega F., Ubago-Jiménez J.L., Puertas-Molero P., Ramírez-Granizo I.A., Muros J.J., González-Valero G. (2020). Effects of an Alternative Sports Program Using Kin-Ball in Individuals with Intellectual Disabilities. Int. J. Environ. Res. Public Health.

